# Meta-review on Perforation Model of Subarachnoid Hemorrhage in Mice: Filament Material as a Possible Moderator of Mortality

**DOI:** 10.1007/s12975-022-01106-4

**Published:** 2022-11-23

**Authors:** Serdar Alpdogan, Timo Sander, Rui Zhang, Dilaware Khan, Xuanchen Li, Huakang Zhou, Ke Li, Ann-Christin Nickel, Baolong Zheng, Anastasiya Skryabin, Simon Schieferdecker, Björn B. Hofmann, Daniel Maximilian Donaldson, Jan Frederick Cornelius, Daniel Hänggi, Sajjad Muhammad

**Affiliations:** Department of Neurosurgery, Medical Faculty, University Hospital Duesseldorf, Heinrich Heine University, Moorenstraße 5, 40225 Duesseldorf, Germany

**Keywords:** Subarachnoid hemorrhage, SAH, Perforation model, Mouse, Mortality

## Abstract

Robust preclinical models are inevitable for researchers to unravel pathomechanisms of subarachnoidal hemorrhage (SAH). For the mouse perforation model of SAH, the goal of this meta-review was the determination of variances in mortality, SAH severity grade, and vasospasm, and their experimental moderators, as many researchers are facing with incomparable results. We searched on the databases *PubMed*, *Embase*, and *Web of Science* for articles describing in vivo experiments using the SAH perforation mouse model and measuring mortality, SAH grade, and/or vasospasm. After screening, 42 articles (total of 1964 mice) were included into systematic review and meta-analysis. Certain model characteristics were insufficiently reported, e.g., perforation location (not reported in six articles), filament (material (*n* = 15) and tip texture (*n* = 25)), mouse age (*n* = 14), and weight (*n* = 10). Used injective anesthetics and location of perforation showed large variation. In a random-effects meta-analysis, the overall animal mortality following SAH was 21.3% [95% CI: 17.5%, 25.7%] and increased with longer observational periods. Filament material significantly correlated with animal mortality (*p* = 0.024) after exclusion of hyperacute studies (time after SAH induction < 24 h). Reported mean SAH grade was 10.7 [9.6, 11.7] on the scale of Sugawara (J Neurosci Methods 167:327–34, 2008). Furthermore, mean diameter of large cerebral arteries after SAH was reduced by 27.6% compared to sham-operated non-SAH mice. Uniforming standards of experimental procedures and their reporting are indispensable to increase overall comparability.

**Supplementary Information:**

The online version contains supplementary material available at 10.1007/s12975-022-01106-4.

## Introduction

Subarachnoid hemorrhage (SAH) is a life-threatening event affecting mainly younger patients compared to ischemic stroke [1]. The acute mortality after aneurysmal SAH is very high. Nearly 15% of the patients die before being hospitalized and only less than 50% of survivors who received adequate professional interdisciplinary treatment are able to return to their previous life. The remaining 50% suffer further complications like neurological deficits, making them dependent on help for the rest of their life with a poor quality of life [2]. To investigate pathophysiology and pathomechanism of this fatal disease, there are several well-studied animal models, which have been used for nearly the last 40 years [3]. Barry et al. developed a rodent model of SAH in 1979 [3] and since then, especially mouse models are commonly used to explore involved molecular pathways of this distinct disease. The reason for using mouse models is the easy and relatively cheap livestock farming and the possibility to generate transgenic animals. Of these, the intravascular filament perforation of an intracranial artery (circle of Willis) [4] (a schematic illustration is added in the supplement section as Fig. S1) and direct blood injection into one of the cisterns (cisterna magna, prechiasmatic cistern) of the brain [5, 6] display the most popular and predominantly used procedures with their specific advantages and disadvantages. On the one hand, the advantage of the injection model is that the SAH blood volume can be controlled [7]. However, it does not mimic acute pathophysiological changes after SAH closely enough. On the other hand, the perforation model has a high mortality rate but displays a more “natural” way of this disease.

In this analysis, we especially focus on the perforation mouse model of SAH to reveal variances of experimental characteristics and factors which might have an impact on mortality rate, SAH grade, and large artery vasospasm with the aim to define a certain standard. A common uniformity in reporting will help to improve comparability of SAH animal model. Additionally, we want to identify conditions which could lower mortality rate resulting in a reduced number of animals needed for in vivo SAH experiments.

## Methods

### Systematic Review

#### Systematic Literature Search

The search for articles using the perforation SAH mouse model was conducted on 19 August 2021 in PubMed, Embase, and Web of Science. The detailed search strategy was (“subarachnoid Hemorrhage” OR SAH OR SAB OR “subarachnoid* bleed*” OR “subarachnoidal Hemorrhage” OR “subarachnoid* Hemorrhaging” OR “subarachnoid* Haemorrhag*”) AND (perforation OR FPM OR cWp) AND (mice OR mouse OR murine). All search results were de-duplicated by two programs described at the end of the “Methods” section. Afterwards, automatic deduplication was manually checked. During screening stages, two reviewers independently screened all articles, while discrepancies between their assessments were resolved by a third independent reviewer.

#### Literature Screening Criteria

To focus on literature relevant for our objectives, we solely included English-language original peer-reviewed research articles describing experiments with the perforation SAH model using wild-type mice. At this, at least one of the following outcomes had to be reported: animal mortality, grade of SAH severity, or large artery vasospasm. Large artery vasospasm reporting was fulfilled if the vessel diameters of either an artery from the circle of Willis or basilar artery of SAH animals in comparison to non-SAH control animals were reported. No other experimental animals or SAH modeling techniques (e.g., blood injection model) were included. The full list of screening criteria is shown in Supplementary Table S2.

#### Data Extraction

For all included articles, 27 experimental data including animal, SAH model, surgical, and outcome-related features were extracted (Supplementary Table S3). Similar to literature screening, data were extracted independently by two reviewers and a third reviewer resolved discrepancies.

### Meta-analysis

#### Exclusions from Meta-analysis

Besides the reporting of usable outcome data already being an inclusion criterion for this review, the number of animals used in an experiment had to be reported for consideration in the meta-analysis. Concretely, mortality rates including the overall number of animals, the grading system for SAH severity, and the corresponding large artery diameter of sham-operated control mice after the same timespan as for SAH-induced mice had to be reported, respectively. However, for meta-regressions and sensitivity analysis, certain articles were excluded. Those exclusions are stated at the appropriate place in this “Methods” section.

#### Meta-analytical Model

We applied a random-effects meta-analytical model [8] as we assumed—and proved through our systematic review of experimental characteristics–true differences between the included experiments. Furthermore, we tested the presence of heterogeneity by using the *Q* statistic. For the computation of true variance of the outcomes (variance caused by differences in experimental design exceeding estimated sampling variance), we used the method described by DerSimonian and Laird [9] as it contains no assumptions regarding the distribution of effects [10].

#### Effect Sizes

For animal mortality, we used the mortality rate (%) as effect size and performed a proportional random-effects meta-analysis. As the mortality rates of SAH perforation models were expected to be around 20% [11], they could not be assumed to be normally distributed [12]. Therefore, mortality rates were transformed via the logit transformation [13] to achieve normal-distributed effect sizes necessary for meta-analysis.

For SAH severity grade, only articles reporting the SAH grade according to the grading system introduced by Sugawara [14] were included, as other grading systems were used too infrequently to undertake a meta-analysis. Thereby, the effect size was the reported mean SAH grade on the used scale from 0 to 18 with the accompanying variance.

For large artery vasospasm, the quotient of arteries’ diameter in SAH perforation mice in comparison to non-SAH sham-operated mice was used as effect size. Thereby, the diameter in the SAH and sham group had to be measured at the same vessel location under equal conditions at the same period after SAH induction to guarantee sufficient comparability.

In case articles reported multiple data for one outcome due to multiple experiments carried out, we summarized the data from the experiments (e.g., calculated overall mortality) if the experiments were performed under equal conditions regarding model parameters and time for outcome measurement. If these conditions were unequal, we included only one of the experiments according to the following rules in order not to violate the condition of independence of data included into meta-analysis:

A If outcome data were presented for different times after SAH induction and for the same animal cohort, then the data with the longest corresponding observational time was included.
B If outcome data were presented for different times after SAH induction and for different animal cohorts, then the experiment with the longest observation period was included into meta-analysis, unless the corresponding number of animals was less than half of the highest number of animals across all experiments. In this case, the experiment with the largest animal cohort was included into meta-analysis, unless its observation period was shorter than 24 h.

We did not note these rules in our ex ante protocol as we did not expect multiple outcome data within studies.

#### Moderator Analysis

If random-effects meta-analysis proved the presence of significant heterogeneity of effects across the studies, then univariate meta-regression was used to reveal possible moderators of either mortality, SAH severity grade, or vasospasm, provided a sufficiently large number of articles report the outcome (at least ten). For categorical parameters, we included articles with non-reported parameter phenotypes with the own category *non-reported*. In case of continuous parameters, we excluded articles with unreported parameter phenotypes to allow linear regression. All conducted meta-regressions are presented in this paper.

Furthermore, we calculated a three-level meta-regression model [15] for the analysis of the influence of the time between vessel perforation and outcome assessment. This three-level model enabled us to include multiple outcomes of an article measured after different periods while upholding the requirement of independence of data included for the meta-analysis. Thereby, we used the restricted maximum likelihood method [16] for the estimation of true variance of effects.

#### Sensitivity Analysis: Exclusion of Hyperacute Studies

Studies measuring outcomes shortly after SAH induction might bias the overall estimation of effects and their moderators. Therefore, we performed a sensitivity analysis with the exclusion of outcomes measured earlier than 24 h after SAH induction and effects measured after an unreported time. We undertook this additional analysis as we think that outcome assessed in these early stages after SAH induction (hyperacute studies) may reflect rather surgery-related causes of death as low experience and skills of the person performing the surgery, than mid-term pathophysiological reasons for fatality like respiratory arrest caused by high intracranial pressure at respiratory area. To avoid the confusion of both leading types of mortality moderators, we see the presentation of separate analyses as a reasonable way to estimate the impact of hyperacute studies. It is important to mention that this sensitivity analysis was not a part of our initial plans stated in the preregistered ex ante protocol as we did not expect such short timespans of mortality assessment after SAH perforation.

#### Software

For the deduplication of systematic search results, we used the deduplication functions of the literature management program Zotero [17] and an online tool developed by the CAMARADES group [18]. Literature screening and data extraction were upheld on the Systematic Review Facility (SyRF) which is especially designed for preclinical animal reviews [19]. Visually presented data were extracted with the WebPlotDigitizer [20]. We conducted all meta-analytical calculations within the RStudio environment [21] and using the programming language R [22]. Within R Studio, the *escalc* and *rma* functions of the R package Metafor [23] were used for the calculation of proportional effect sizes and meta-analysis/meta-regression, respectively. Moreover, the function *forest* of the R package meta [24] was used for the creation of forest plots. The full R code and datasets are available on GitHub (https://github.com/TimoSander/SAH-perforation-model-Review).

## Results

### Systematic Review

#### Systematic Literature Search

**Fig. 1:**
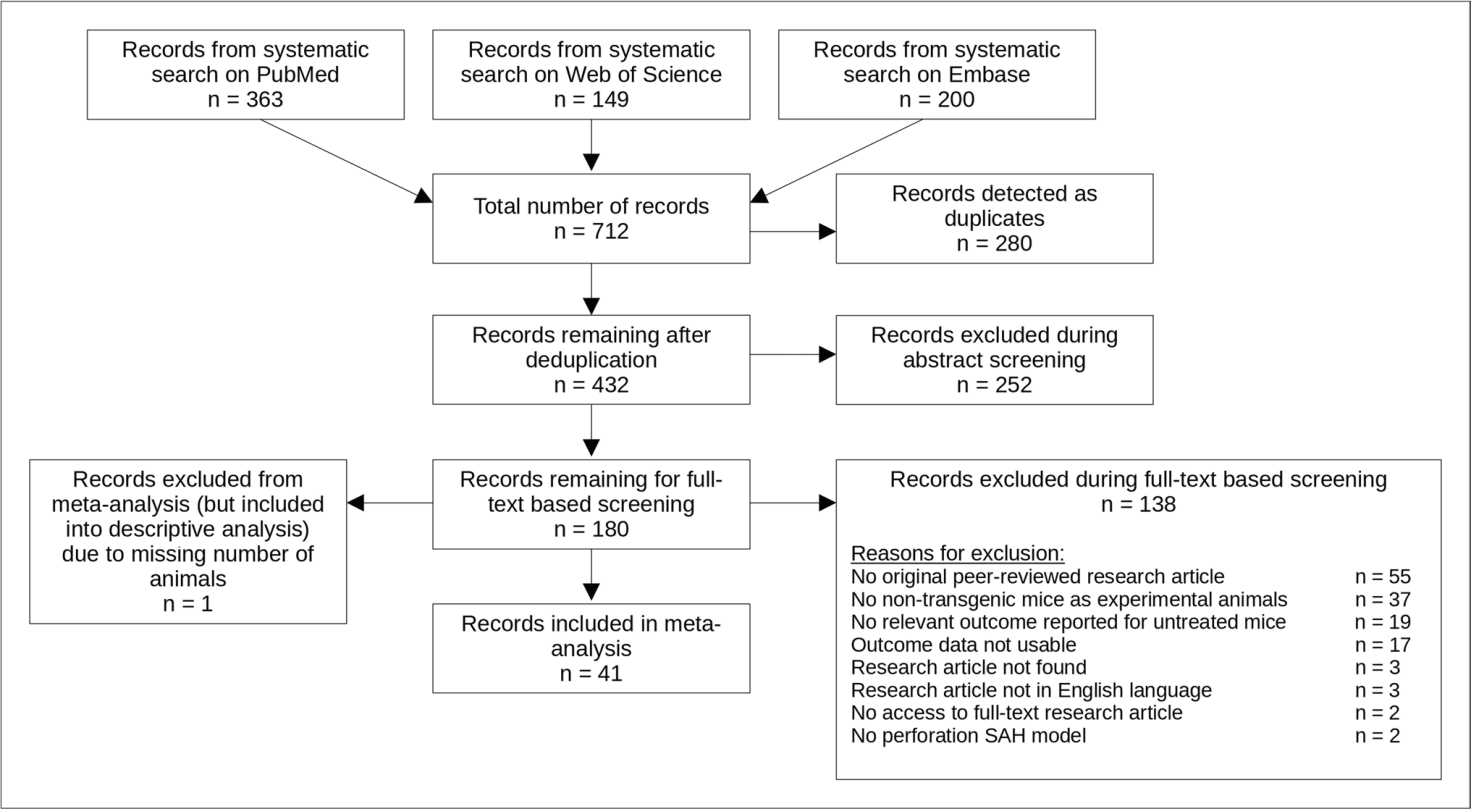
Systematic search literature flow chart. Literature flow chart based on the PRISMA statement 2021 [25]. Systematic search was performed in August 2021. One reason for exclusion per excluded article. SAH, subarachnoid hemorrhage

As shown in Fig. 1, the systemic search on PubMed, Embase, and Web of Science for literature using the SAH perforation model delivered a total of 712 records of which 432 unique articles remained after deduplication. After abstract and full-text screening, 42 articles were included into descriptive analysis of perforation model parameters. However, one article was excluded from the subsequent meta-analysis due to an unreported number of experimental animals. During full-text screening, the most frequent reasons for exclusion were publication types different from peer-reviewed research articles (*n* = 55) as well as other experimental animals used than mice (mostly rats, *n* = 37). In addition to that, we could not include results of seventeen articles due to unclear group allocation or cumulative SAH group mortalities for different interventional cohorts, making their outcome data unusable for meta-analysis. A table showing all articles with extracted outcome data included into meta-analysis is available as supplementary material (S4).

#### Extracted Methodological Parameters

Overall, a broad diversity in parameters of SAH perforation model experiments was observed, as shown in Table 1.

**Table 1:** Distribution of SAH perforation model experimental characteristics

Mice as experimental animal			SAH induction surgery	
Number of mice (all articles)		Mice (articles)	Filament entry point	Articles
Total (including all outcomes)		1964 (41)	External carotid artery (ECA)	36
SAH-perforation surgery		1435 (41)	Internal carotid artery (ICA)	3
Sham-operation (no SAH)		529 (33)	Middle cerebral artery (MCA)	1
Male		1909 (42)	Location of perforation	Articles
Female		33 (2)	Bifurcation of ICA/MCA	27
Strain		Articles	Anterior cerebral arterial (ACA)	4
C57/BL6 family		42	Internal carotid artery (ICA)	3
C57/BL6		22	Circle of Willis	2
C57/BL6J		17	Duration of surgery	Articles
C57/BL6N		2	15 min	1
C57/BL6N (and ddY)		1	30 min	1
Animal housing conditions			Monitoring of ICP	Articles
Environment		Articles	Yes	10
Single cage		2	No	1
Group cage		4	Postop, stress-management	Articles
12-h light–dark cycle		16	Methods reported	9
Free access to food and water		22	Filament for perforation	
Temperature		Article	Filament diameter	Articles
Controlled		12	4–0	6
Mean	21 °C	1	5–0	31
	22 °C	4	6–0	1
	23 °C	1	Length	Articles
	24 °C	1	20 mm	2
	25 °C	1	10 mm	1
Humidity		Articles	Material	Articles
Controlled		12	Nylon	22
Mean	30%	1	Prolene	5
	55%	1	Tip	Articles
	60%	1	Sharpened	8
	65%	1	Blunted	9
Anesthesia			Filament composition	
Type		Articles	Monofilament	11
Inhalation		15	Suture	6
Injection		23	Monofilament and suture	16
Inhalation and Injection		1	Outcome measurements	
Injective anesthesia drugs		Articles	SAH severity grading system	Articles
Pentobarbital		9	Sugawara et al. (2008)	16
Tribromoethanol		5	Egashira et al. (2015)	2
Midazolam, medetomidine, fentanyl		4	Parra et al. (2002)	1
Ketamine, xylazine		4	Use of drug vehicle control	Articles
Chloral hydrate		1	Yes	31
Injective anesthesia drugs		Articles	No	11
Isoflurane		15	Outcome type for meta-analysis	Articles
Intubation of mice		Articles	Animal mortality	41
Yes		5	SAH severity	19
No		6	Vasospasm	6

Three articles used female and male mice, and one of them did not report the exact distribution of male and female. If the sum of articles for all phenotypes of an article does not match the total number of relevant articles, then the remaining number of articles did not report the parameter. *ddY*, Deutschland, Denken, and Yoken; *ICP*, intracerebral pressure; *Fent*., fentanyl; *Postop*., postoperative; *SAH*, subarachnoid hemorrhage.

#### Experimental Animals and Housing Conditions

The total number of mice included in this review was 1964, of which 1435 received SAH perforation and 529 animals underwent a sham-operation without perforating an intracranial artery. Only 33 of them were female. Almost all mice were from C57/BL6 strain. Only one article used a strain called ddY (Deutschland, Denken, and Yoken), which has been established as an inbred strain from the ddY colony at Yoken, which is presently the National Institute of Infectious Diseases in Japan. However, there was no outcome of interest for the ddY mice reported in this article. Mice mean age was 11.8 weeks (ranged from 7 to 32 weeks), while mice mean weight was 24.3 g (ranged from 20 to 31.9 g). Regarding animal housing conditions, free access to food and water as well as 12-h light–dark cycles represented frequently reported environmental settings. Interestingly, of the articles that reported numbers of mice per cage, group cage was more often present than single cage housing. Concerning temperature and humidity, many authors reported that they controlled these conditions but did not tell the exact numbers.

#### Anesthesia and Surgery

Injective anesthesia (administered intraperitoneally) was the most widely used way to anesthetize mice. Thereby, five different anesthetic substances (or combinations) were chosen. In case of inhalative administration, isoflurane was the only drug used. In addition to that, mice were intubated in five articles. For induction of SAH via filament perforation, we observed serious variances in the location of perforation. As 27 articles reported filament perforation at the bifurcation of the internal carotid artery (ICA) and middle cerebral artery (MCA), less authors chose the anterior cerebral artery (ACA) and ICA or just described the location of perforation as circle of Willis without further description. Concerning the entry point of the filament into the cerebrovascular system, the choice was more uniform with 36 studies introducing the filament via the external carotid artery (ECA) and only three studies via ICA and one through the MCA. Surprisingly, intraoperative intracranial pressure measurement was reported in only ten articles. After the operation, nine authors reported special pain-/stress-management actions with treatment with buprenorphine and carprofen as the most frequently used analgesics.

#### Filament for Perforation

The diameter of the filament used for vessel perforation was mainly 5–0, but 4–0 and 6–0 filaments were used, too. Major differences occurred in the material and tip texture, as a considerable number of articles reported nylon and prolene filaments as well as sharpened and blunted filament tips, respectively. Interestingly, sixteen authors described their filament texture as both monofilament and suture at the same time.

#### Outcomes

Forty-one articles reported animal mortality, nineteen SAH severity score, and six large artery vasospasm. The most frequent times of mortality reporting were 24 h (22 articles), 48 h (16 articles), and 72 h (14 articles) after SAH induction. The longest timespan was 30 days between SAH induction/perforation and mortality assessment (two articles). Similarly, the SAH severity score assessment was most often performed 24 h after SAH induction (13 articles). Thereby, three different grading systems were used: 16 studies applied the one described by Sugawara [14], two studies the one by Egashira [26], and one article in accordance to the system introduced by Parra [27]. Large artery vasospasm was measured in only six studies. Interestingly, eleven studies reported the use of a drug vehicle control with phosphate-buffered saline (PBS), NaCl, DMSO (dimethyl sulfoxide), or ethanol.

#### Reporting of Methodological Parameters

**Fig. 2:**
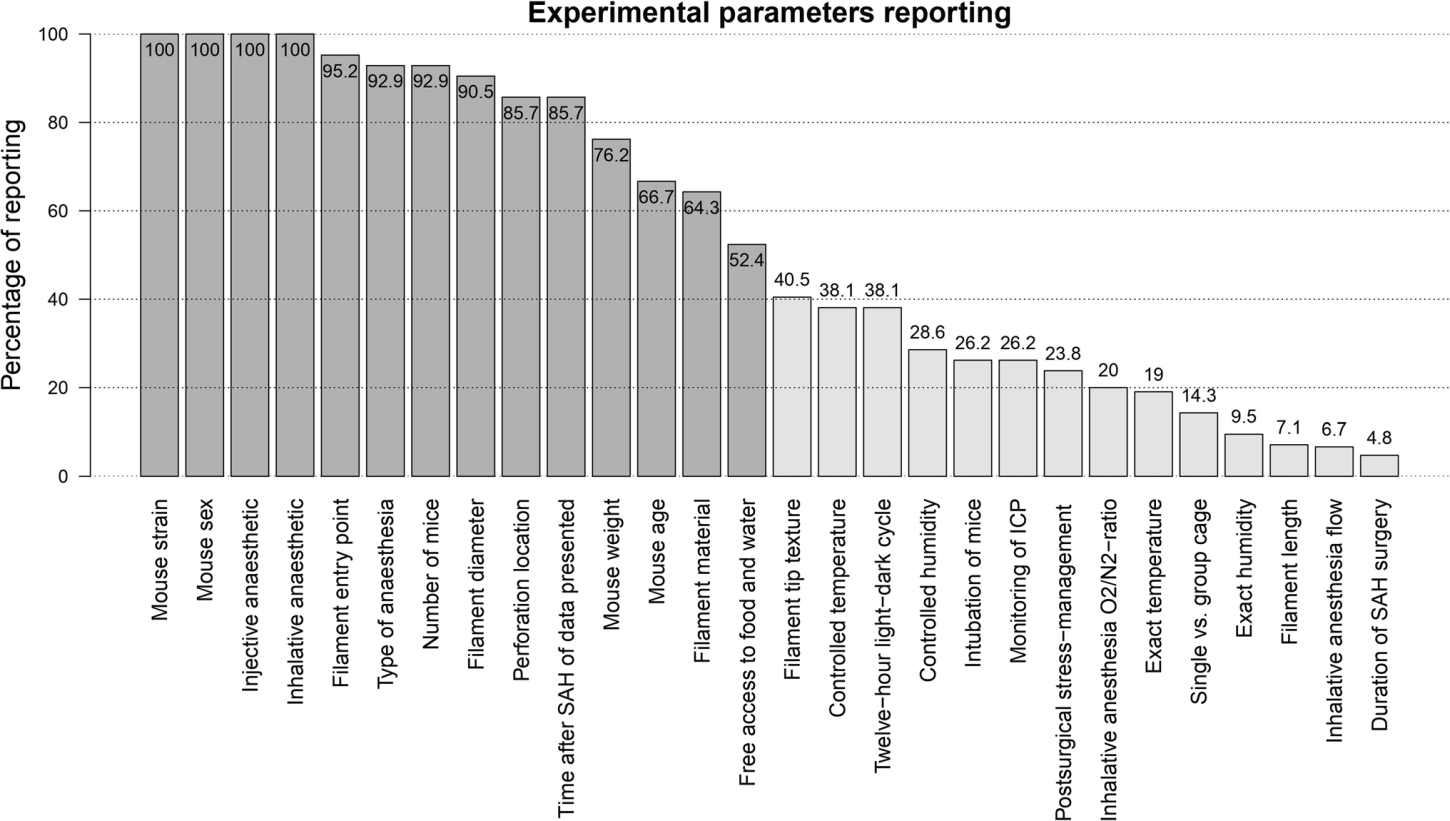
Reporting of experimental parameters. Share of articles that reported the parameters. For injective and inhalative anesthesia parameters, the referring total number of articles was reduced due to the applicability of the parameters depending on type of anesthesia. Outcome-related parameters (e.g., time after SAH, number of mice) were considered reported if they were reported for every relevant experiment in an article. ICP, intracranial pressure; SAH, Subarachnoid hemorrhage

The frequency of reporting of parameters relevant for the replication and interpretation of experimental results showed large inconsistencies, as shown in Fig. 2. Concerning, even several key features of the SAH perforation model were unreported in a non-negligible number of articles.

These included filament entry point (reported in 40 of 42 articles (95.2% of all articles)) and location of SAH perforation (85.7%), as well as filament diameter (90.5%) and material (64.3%). Furthermore, crucial parameters for the interpretation of results were missing in a notable number of studies, e.g., number of animals (reported in only 92.9% of the articles) and time after SAH perforation (85.7%). Moreover, weight and age of experimental mice were unclear in ten and fourteen articles, respectively. Regarding environmental parameters, the exact temperature and humidity were described in just 19.0% and 9.5% of the articles, respectively, while the grouping of animals in single or cohort cages was similarly seldom reported (14.3).

### Meta-analysis

#### Overall Mouse Mortality

**Fig. 3:**
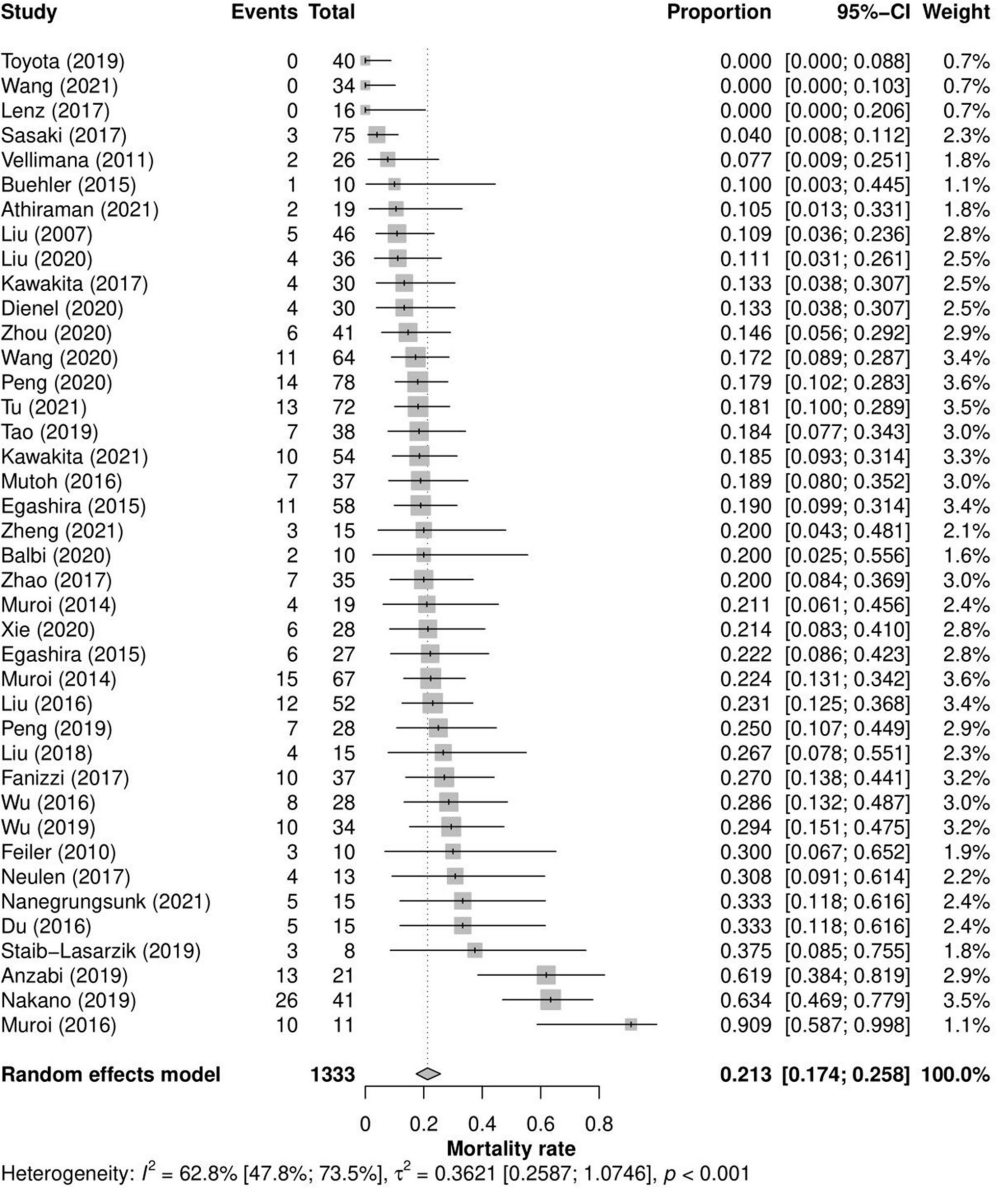
Mouse mortality forest plot. The columns “Events” and “Total” describe the number of dead animals and total experimental animals, respectively. The column “Proportion” indicates mortality rates. The dotted vertical line represents the overall random-effects weighted mean mouse mortality. The article effects are weighted by the inverse of their variances. CI, confidence interval

Across 40 articles that provide sufficient data to be included in meta-analysis, the overall weighted mean animal mortality after SAH modeled through filament perforation was 21.3% [95% CI: 17.4%, 25.8%]. As shown in Fig. 3, this mortality showed significant and meaningful heterogeneity caused by true differences across the articles (*p* < 0.001), represented in a very broad 95% prediction interval of mouse mortality in a potential future experiment from 7.5 to 47.5%. This heterogeneity made up 62.8% of the total variance observed (remaining share explained by sampling variance). Regarding sham-operated mice, the overall weighted mean mortality across 29 articles including 499 mice was 5.1% [3.3%, 7.9%]. Thereby, no mice died in 24 of the 29 articles and the maximum share of dead mice after sham-operation was 23.1% in a study where two of nine mice died. As the test for heterogeneity of sham-operated mouse mortality across the studies was non-significant (*p* = 0.940), moderator analysis was omitted for this group.

#### Moderators of Mouse Mortality

**Fig. 4:**
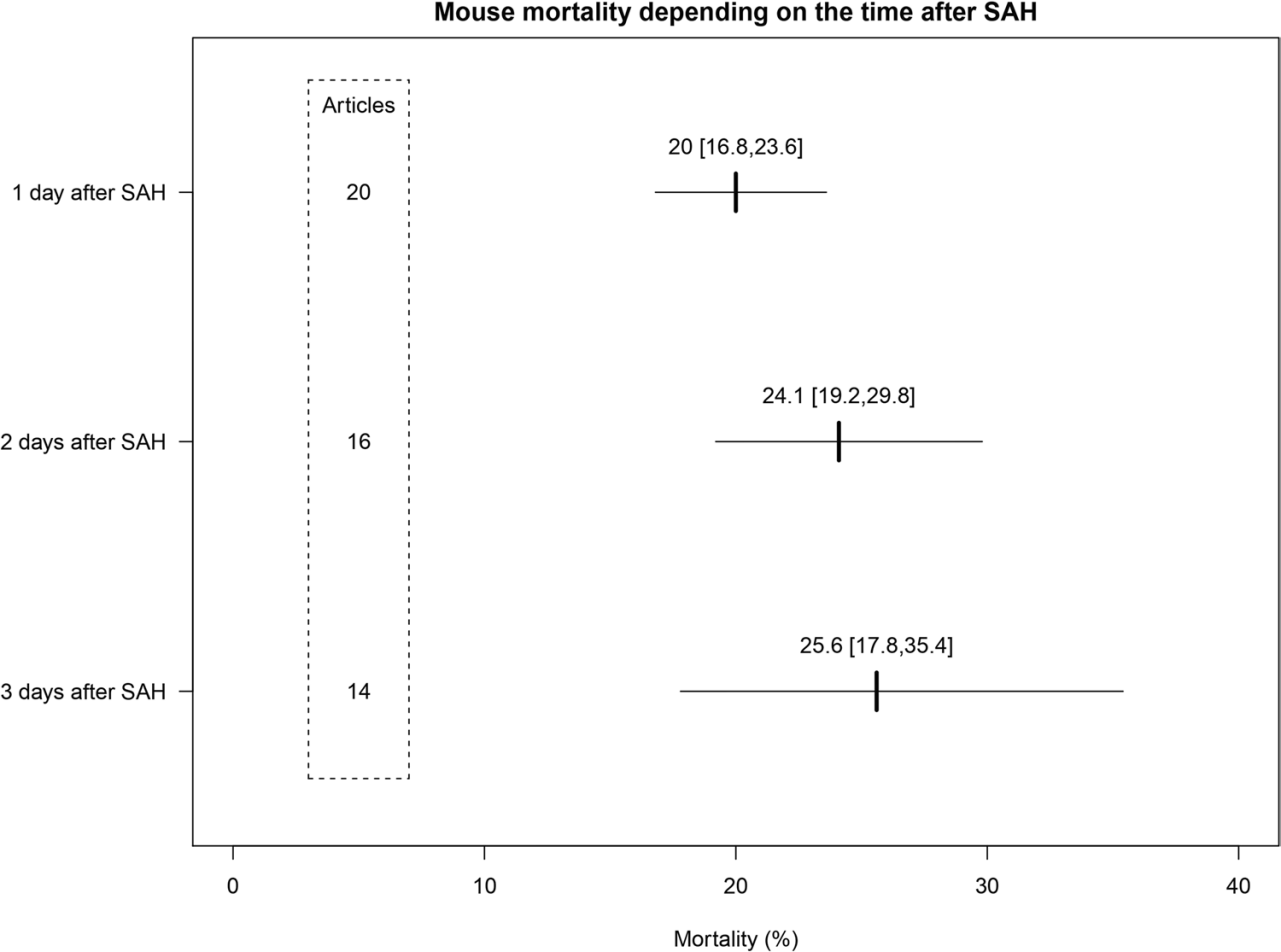
Mouse mortality at different timespans after SAH perforation. Cumulative weighted mean mortality estimates of random-effects meta-analyses for three subgroups divided by time after SAH induction. Confidence intervals (95%) are represented by horizontal line width and reported in brackets. Values in dotted box show the number of articles in the subgroup meta-analysis that reported mouse mortality at the indicated time after SAH. SAH, subarachnoid hemorrhage

In univariate meta-regressions shown in Table 2, the time after SAH perforation induction was the only significant moderator of animal mortality (*p* = 0.002). As shown in Fig. 4, the mortality 24 h after SAH was 20.0% [16.8%, 23.6%] and increased by 4.1 to 24.1% [19.2%, 29.8%] 2 days after SAH perforation. Three days after SAH induction, a total of 25.6% [17.8%, 35.4%] of the mice died cumulatively.

**Table 2:** Moderators of mouse mortality: univariate meta-regressions

Parameter	*p* value	Marginal *R*^2	Residual *I*^2
12-h light–dark cycle	0.343	-	-
Free access to food and water	0.233	-	-
Controlled temperature	0.479	-	-
Controlled humidity	0.433	-	-
Type of anesthesia	0.163	-	-
Injective anesthesia drugs	0.486	-	-
Intubation of mice	0.514	-	-
Location of perforation	0.694	-	-
Monitoring of ICP	0.289	-	-
Postop. stress-management	0.424	-	-
Filament diameter	0.566	-	-
Filament material	0.103	-	-
Filament tip	0.728	-	-
Description of filament texture	0.136	-	-
Vehicle control	0.662	-	-
Time after SAH perforation	0.002*	14.4%	52.0%

The column “Marginal *R*^2” indicates the moderator fit according to Nakagawa (2013) and residual *I*^2 the remaining across-studies true variance after inclusion of the particular moderators. *Significant moderation of mortality in a three-level meta-regression at significance level alpha = 0.05.

#### Sensitivity Analysis: Exclusion of Hyperacute Studies

For a sensitivity analysis that may add useful insights into mouse mortality, we removed four studies that solely presented data for hyperacute experiments (which we defined as an observational time less than 24 h following SAH perforation). Moreover, we excluded two articles that did not reported the time after SAH of mortality assessment. As shown in Table 3, the overall mortality estimate increased slightly to 23.4% [95% CI: 19.3%, 28.0%] while the presence of heterogeneity across the articles stayed almost unchanged (*I*^2 = 59.0%).

**Table 3:** Sensitivity analysis of mouse mortality: exclusion of hyperacute studies

Number of included articles	34
Overall weighted mean mouse mortality	23.4% [19.3%, 28.0%]
Test for heterogeneity of effects (*Q* statistic)	*p* val. < 0.001
Tau^2 (estimated variance of effects)	0.2864 [0.1171, 0.8413]
*I*^2 (share of variance of true effects in total variance	59.0% [37.1%, 80.9%]
Prediction interval (for a potential future studies effect)	9.4–47.3%

Values in brackets represent 95% CI. Note that tau^2 is presented in inverse logit transformation

Testing filament material as the only parameter close to significant moderation of mortality in the meta-regression including all data, exclusion of hyperacute studies resulted in an improved fit of this univariate meta-regression model (*p* = 0.024). Thereby, the reporting and type of filament material accounted for 20.0% of the heterogeneity (*R*^2) across the articles and the remaining share of heterogeneity (*I*^2) decreased to 53.1% [95% CI: 25.1%, 77.2%]. As shown in Table 4, the main difference lies between unreported filament material and nylon with a 73% higher mortality in articles where the filament material for perforation was not specified. Interestingly, there was no significant difference between nylon and prolene as filament material. Importantly, a biased result for filament material estimates may be possible as the observation period after SAH induction was slightly longer in the unreported cohort.

**Table 4:** Correlation between mouse mortality and filament mortality (excluding hyperacute studies)

Filament material univariate meta-regression excluding hyperacute studies (*t* < 1 d)				
*p* value (test of moderator)				0.0240
*R*^2 (fit of moderator)				20.0%
Residual *I*^2 (estimated share of true across-studies variance)				53.1% [25.1%, 77.2%]
Filament material	Articles	Mouse mortality	*p* value	Mean time (days)
Nylon	19	19.2% [15.0%, 24.3%]	0.007	4.1 (SD = 6.6)
Prolene	5	25.0% [15.8%, 37.2%]	0.269	2.2 (SD = 1.1)
Unreported	10	33.4% [24.3%, 44.0%]	0.002	7.4 (SD = 8.3)

The column “Mean time” indicates the mean time after SAH induction in the studies with the particular filament materials. Values in square brackets represent 95% CI. Hyperacute results (observational period < 24 h) were excluded.

#### SAH Severity Grade

**Fig. 5:**
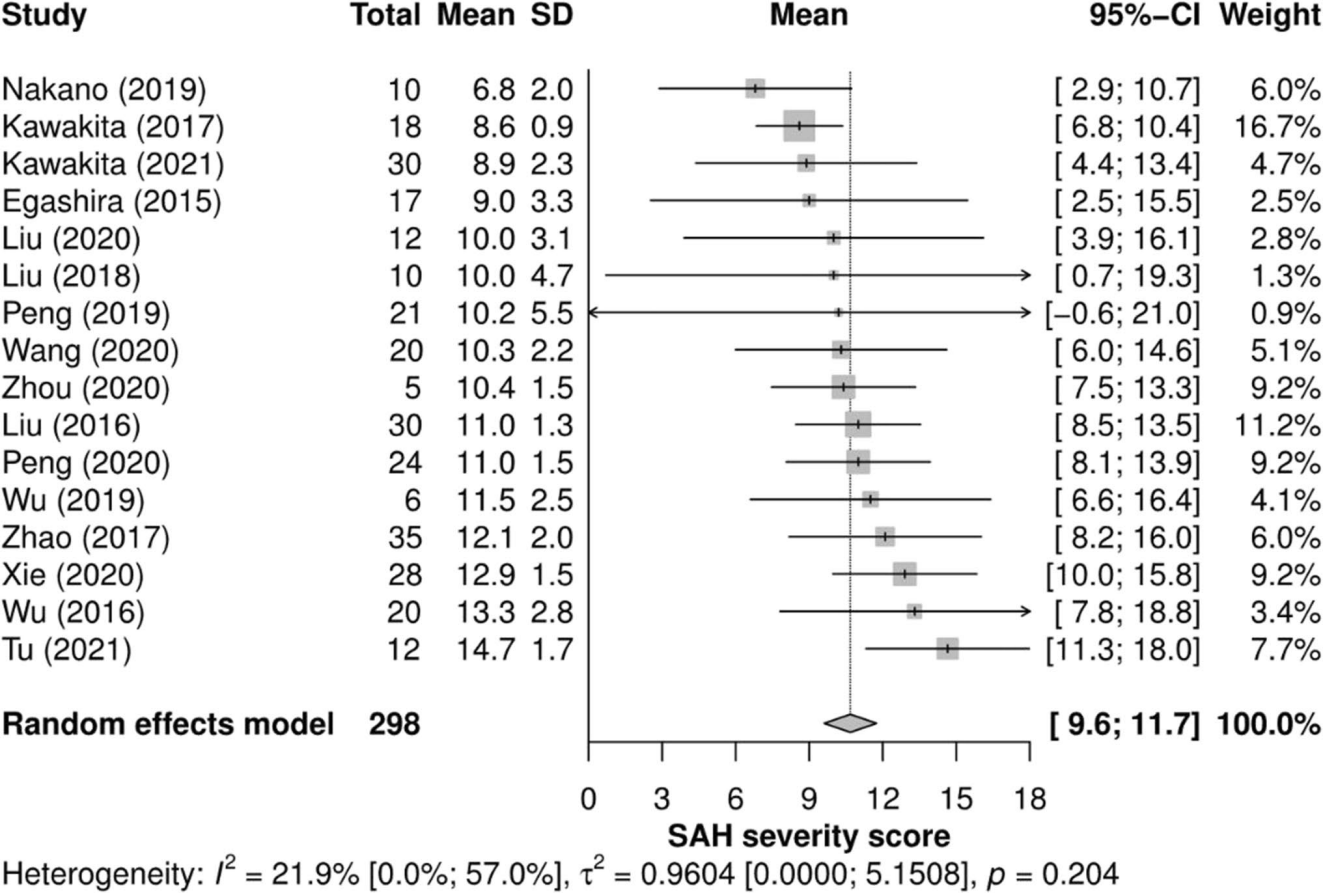
SAH severity grade forest plot. Random-effects meta-analysis. The column “Total” shows the number of mice per article subjected to SAH grading. The column “Mean” represents the SAH severity score reported in the articles. Only SAH severity scores according to the grading system introduced by Sugawara (2008) were included. SAH, subarachnoid hemorrhage

Including 16 articles and 298 mice that reported a score for SAH severity in accordance to Sugawara [14], the overall weighted mean SAH grade was 10.7 [95% CI: 9.6, 11.7], as shown in Fig. 5, and indicates moderate SAH on the scale of Sugawara (13). There was no significance in the test for heterogeneity across the studies (*p* = 0.204) and therefore, we conducted no subsequent moderator analysis. Only one study described mild (score of 0–7) and two studies described severe SAH (score of 13–18). Regarding sham-operated mice, ten authors reported SAH grade in accordance to Sugawara [14]. At this, the score 0 was described in eight articles while two reported a score of approximately 2. As mice were sham-operated without vessel perforation, a score of 0 was expected for the sham group and therefore, no meta-analysis of their scores was performed.

#### Large Artery Vasospasm

Six articles reported the measurement of vasospasm in large cerebral arteries including ICA, MCA, ACA, and basilar artery, but one of them was excluded from further analysis due to non-reporting of the number of animals. Because of the limited number of studies reporting vasospasm, we decided to omit performing meta-analysis and instead present the results of each article in Table 5. The largest vasospasm in SAH-induced mice was measured with 49.9% artery diameter in comparison to the corresponding sham-operated mice artery diameter. The overall vasospasm across the five studies was 72.4% (weighted by number of mice). Only one article allowed to observe differences between ipsi- and contralateral side of vessel perforation where the vasospasm was substantially greater on the side of perforation.

**Table 5:** Large artery vasospasm after SAH in perforation model

Study	Mice	Time (days)	Vessel	Vasospasm (SD)
Athiraman (2021)				**69.6% (22.0%)**
	17	3	Left MCA	69.6% (22.0%)
Jayaraman (2021)				**74.2% (19.5%)**
	15	3	MCA	74.2% (19.5%)
Vellimana (2011)				**73.4% (24.0%)**
	16	2	MCA	73.4% (24.0%)
Kawakita (2017)				**49.9% (13.6%)**
	7	1	Right ACA (contralateral)	61.4% (17.6%)
	7	1	Left ACA (ipsilateral)	27.6% (7.6%)
	7	1	Right MCA (contralateral)	57.2% (16.1%)
	7	1	Left MCA (ipsilateral)	27.0% (8.0%)
	7	1	Right ICA (contralateral)	56.1% (17.3%)
	7	1	Left ICA (ipsilateral)	31.0% (12.7%)
	7	1	Basilar artery	89.0% (11.7%)
Dienel (2020)				**90.4% (9.2%)**
	9	5.7	Right ACA	83.6% (8.0%)
	9	5.7	Left ACA	96.0% (9.8%)
	9	5.7	Right MCA	84.7% (7.4%)
	9	5.7	Left MCA	87.0% (12.5%)
	9	5.7	Right ICA	94.8% (5.2%)
	9	5.7	Left ICA	90.4% (13.1%)
	9	5.7	Basilar artery	96.5% (4.5%)
Total	64			**72.4% (22.3%)**

Vasospasm is presented as proportion of artery diameters to corresponding sham-operated mice artery diameters (set to 100%). In case articles reported multiple vasospasm data, the mean vasospasm in this study was calculated and is presented in the first row of each article. If vasospasm measurement side in comparison to SAH perforation side was reported, it is stated with contra- and ipsilateral. *ACA*, anterior cerebral artery; *ICA*, internal carotid artery; *MCA*, middle cerebral artery; *SAH*, subarachnoid hemorrhage

## Discussion

### Heterogeneity of Animal Mortality and Moderating Factors

Our analysis revealed significant and meaningful heterogeneity of mouse mortality after SAH induction using the filament perforation model. This heterogeneity was accompanied by large inconsistencies in perforation model parameters. These included missing standards for the filament type (suture or nylon) and shape (sharpened, blunted) used for the perforation, and the entry point as well as for the exact location of the perforation. A non-negligible number of articles even did not report these key features. However, there was no significant moderation of mortality for any of the assessed experimental parameters except the time after SAH induction.

Because of the differences of times of mortality reporting after SAH, we conducted a sensitivity analysis excluding hyperacute mortality data (*t* < 24 h) as we expected different main causes of death in early and mid-term stages after SAH. In this additional analysis, we observed a significant correlation of animal mortality with the material of the filament used for perforation. Thereby, the mortality in the articles with unreported material was linked with a higher mortality than in the articles using nylon filaments. However, we are careful to derive direct conclusions regarding a recommended filament material due to multiple reasons. First, there was no significant difference of mouse mortality between the two reported materials nylon and prolene. Second, the exclusion of hyperacute mortality data should be recognized as an additional investigation and was not planned in advance for this review. Third, this correlation may be biased due to average longer observation periods in the cohort of articles with unreported filament material.

The presented meta-analysis could not identify significant mortality moderation through experimental characteristics that theoretically should affect the death rate; e.g., a sharpened filament will probably puncture the vessel more accurately and precisely than a blunted filament that might roughly pierce the vessel wall, thus causing a bigger defect in the vessel, increased bleeding volume and ICP, and subsequently increase mortality. A leading reason for these analytical difficulties could be the presence of soft parameters that are difficult to measure and therefore were not reported in the articles, although they are likely to contribute hugely to death rates. Such parameters may be present in varying surgical experience, for example, if the model is already well established and performed on a regular basis in the group or if they just started establishment process, or individual skills of each experimenter, updated laboratory equipment (different monitoring devices), and even the procedure itself allowing plenty of possibilities for model inconsistencies across the studies. A solution to overcome this problem may oppose the analysis of mortality across multiple studies published by the same laboratory group, but this approach seems to be impaired as laboratory groups tend to adhere to established materials and practices not offering enough variability for moderator analysis.

Improved reporting practices of key experimental elements may help to enhance the strength of explorative reproducibility moderator analyses in the future, not least in a scientific environment that increasingly promotes the importance of reproducible findings for scientific advance. Currently, the non-reporting of certain methodological features may be a result of word restrictions in publications and non-reported standards, which may be self-evident for researchers in the special field, but not for external readers. Despite being included in the widely recognized reporting guideline for in vivo studies ARRIVE [28], many articles did not adhere to it, providing insufficient details of experimental animals, which was also observed by other reviews [29, 30]. Furthermore, authors should keep certain standards for comparability regarding environmental conditions including temperature and humidity as well as if animals were housed separately or in groups. This might have an impact on recovery whether due to missing social interactions or due to increased cage fights.

### Heterogeneity of SAH Grade and Vasospasm

Positively, the overwhelming majority of studies that reported a SAH grade adhered to the scoring system introduced by Sugawara et al. [14], allowing comparability of SAH severity across studies. Moreover, we found no evidence for heterogeneity of SAH scores across the included articles, enhancing comparability, too. This homogeneity might base on the exclusion of mild SAH mice described by many authors. Regarding large artery vasospasm, the low number of articles in this review reporting this outcome made it difficult to derive conclusions on the overall reproducibility, but the trend indicated a mean artery diameter after SAH induction of 70% compared to no sham-operated non-SAH mice. As one study showed the relevance of the side of diameter measurement in relation to the side of SAH perforation [31], enhanced reporting of this parameter should be reported for evaluability of results.

### Transferability of Model Results into Clinical Practice

Animal models are essential to understand and study pathomechanisms of SAH and to find proper treatment or a potential cure. The overall goal of a 1:1 direct transferability into clinical practice is discussed controversially regarding complex interplay of inflammation in the physiology [32, 33] of disease. Thus, it is important to mimic the human characteristics of the disease as close as possible. Regarding experimental animals, high costs and small animal numbers have forced to switch specimen from large animals such as primates [34] and dogs to small-sized animals like rats and mice over the last decades [3, 35]. With the use of rodents, generating transgenic animals for different diseases and using large experimental sizes became easier due to simple animal husbandry, handling, and lower costs. Although, important experimental animal characteristics do not reflect epidemiology of SAH in human; e.g., male mice are predominantly used in SAH model, despite the observation of a higher incidence in women [1]. Furthermore, the mice used as experimental animal in this review had a mean age of 11.8 weeks, which represents an early stage of their life as C57/BL6 mice are considered middle-aged or old at 10–14 and 18–24 months, respectively [36]. In contrast, incidence of SAH in humans constantly increases with rising ages [37]. Despite increased costs, mice in at least middle age may model cerebrovascular aging process leading to SAH more realistic.

Despite the preferred use of the perforation model to study SAH in mice [35], there are advantages and disadvantages in comparison to other in vivo models. The injection model allows to control initiation time, total blood volume, and thus rate of hemorrhage. On the contrary, vasospasms mimic close to reality in the perforation model, but blood volume and size of the affected area cannot be controlled directly. Besides that, the main disadvantage is that spontaneous rupture of intracranial vessels which occurs in clinical cases cannot be observed and thus, early and delayed mechanisms of SAH cannot be studied properly, offering a difficulty in translation from bench to bedside. Gruiter and his colleagues [38] conducted a review of in vivo SAH modeling practices which showed that infrequent reporting of experimental parameters is not only a problem of the filament perforation model, but also of other techniques used for SAH induction. No model was superior, so none of them can be especially recommended from the point of view of comparability and reproducibility. For the authors of SAH —in vivo studies, it is of particular importance to report on the conditions they have chosen, regardless of which model they used.

As we emphasize that the standardization of experimental characteristics of the filament perforation SAH model is urgently needed for enhanced comparability and reproducibility of results on that model, the question rises what the exact standards for that model should be. However, this review does not advice concrete model parameters for laboratory practice because of the limited body of data, as the review proved, not providing sufficient substance for such standards. To find and establish concrete standards, authors need to report their used model parameters to be able to derive evidence-based recommendations for optimal experimental parameters in terms of mortality, SAH modeling, and reproducibility. In the end, more frequent reporting of experimental conditions can enhance the comparison of their fit and accelerate the process of finding standards.

### Additional Comments on Own Practical Experience

We are successfully using the filament perforation model for SAH induction in our lab and are happy to share our own experience as we have faced major difficulties in reproducing results of other groups during the establishment process.

We predominantly use adult (12–16 weeks) male mice to make sure that size and weight fit optimal for the previously reviewed articles. They were anesthetized with isoflurane, and blood flow and intracranial pressure were monitored to check successful perforation. Out of the three different filament sizes and different textures reported in the articles, uncoated 5–0 nylon monofilament was the one which showed to be the best effective choice in our own laboratory.

## Conclusion

Our work demonstrates that a uniform and standardized methodology is urgently needed to compare results of different researchers on the same neurovascular disease model all over the world. Uniformed standards of reporting will lead to improve comparability and thus, a reduction of animal numbers used in this model can be achieved in accordance to the principles of 3 R.

### Supplementary Information

Below is the link to the electronic supplementary material.

Supplementary file1 (JPG 135 KB)

Supplementary file2 (PDF 637 KB)

## Data Availability

The full statistical code and its underlying raw data are publicly available on GitHub (https://github.com/TimoSander/SAH-perforation-model-Review).

## Declarations

This article includes studies with animals which were not performed by any of the authors.

This review follows the PRISMA guideline on transparent reporting of a meta-review.

### Open Access

An ex ante protocol published before the conduction of the meta-analysis is publicly available at the Open Science Framework (https://osf.io/gey2k/).
