# Supplementary figures and images for: Meta-review on Perforation Model of Subarachnoid Hemorrhage in Mice: Filament Material as a Possible Moderator of Mortality
Source: Transl Stroke Res. 2022 Nov 23;15(1):16–29. doi: 10.1007/s12975-022-01106-4 (PMC10796476; doi:10.1007/s12975-022-01106-4)

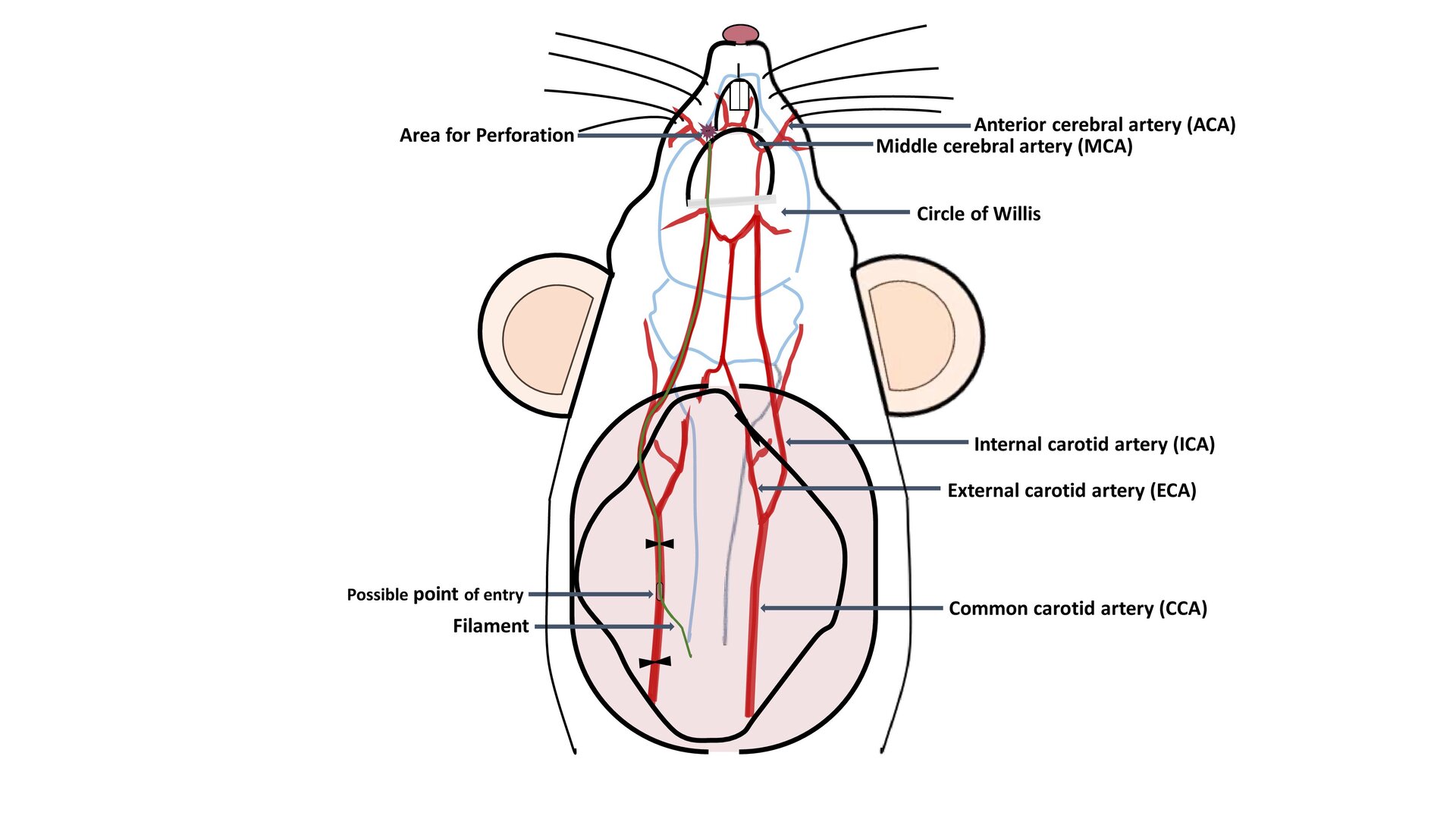

Supplement: Supplementary file 1 — Supplementary file1 (JPG 135 KB) [file 12975_2022_1106_MOESM1_ESM.jpg]
