# Supplementary material for: Meta-review on Perforation Model of Subarachnoid Hemorrhage in Mice: Filament Material as a Possible Moderator of Mortality
Source: Transl Stroke Res. 2022 Nov 23;15(1):16–29. doi: 10.1007/s12975-022-01106-4 (PMC10796476; doi:10.1007/s12975-022-01106-4)
Supplement: Supplementary file 2 — Supplementary file2 (PDF 637 KB) [file 12975_2022_1106_MOESM2_ESM.pdf]

Supplementary material

S1: Schematic illustration of filament perforation model in mouse

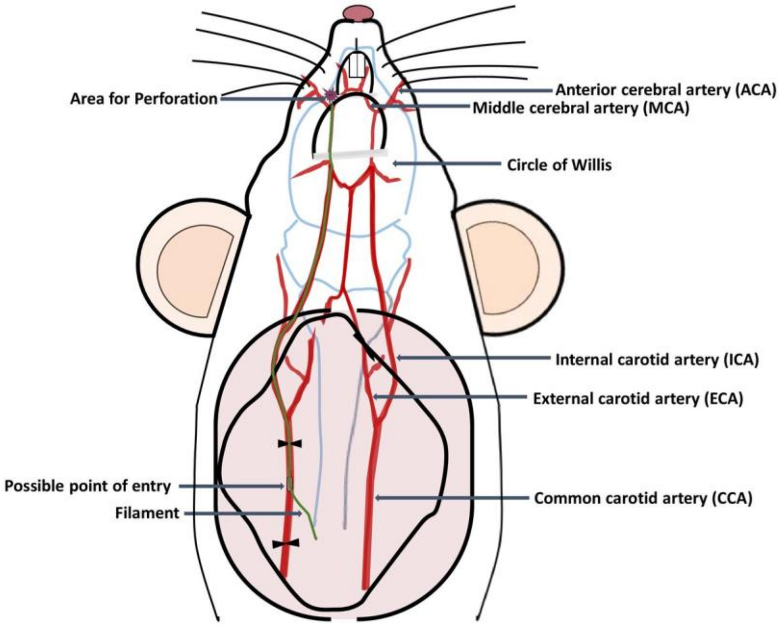

Supplement 1: The nylon monofilament (green line) is inserted via common carotid artery (CCA) and forwarded via the internal carotid artery (ICA) to the bifurcation of middle cerebral artery (MCA) and anterior cerebral artery (ACA). The vessel wall is punctured with the filament to cause the bleeding into the subarachnoid space.

S2: Literature screening criteria

Inclusion criteria	Exclusion criteria
SAH perforation model	Other SAH models (e.g., injection model)
Wild-type mice as experimental animals	Other animals (e.g., rats)<br>Genetically modified mice
Reporting of at least one outcome:<br>animal mortality, SAH severity grade, large artery vasospasm	None of the outcomes reported
Original peer-reviewed research articles	Other publication types (e.g., conference abstracts, book chapters)
English language	Other languages than English

Supplement 2: All inclusion criteria had to be fulfilled for an article to be included in the review. SAH = Subarachnoid haemorrhage.

S3: Extracted parameters

Category	Parameters
Mice as experimental animals	Number<br>Strain<br>Sex<br>Age<br>Weight
Animal housing conditions	Single cage vs. group cage<br>Temperature<br>Humidity<br>Twelve-hour light-dark-cycle<br>Free access to food and water
Anaesthesia	Inhalative or injective anaesthesia<br>Inhalative/Injective anaesthetic drugs<br>Inhalative anaesthesia O2/N2-ratio and flow<br>Intubation
Surgery	Perforation entry point<br>Location of perforation<br>Duration of SAH perforation surgery<br>Monitoring of ICP<br>Postsurgical pain/stress-management
Filament for perforation	Diameter<br>Length<br>Material<br>Tip (sharpened vs. blunted)<br>Texture: monofilament vs. suture
Outcome	Mortality rate, SAH severity grade, large artery vasospasm<br>Timespan after SAH perforation<br>Vehicle controls

Supplement 3: ICP = Intracranial pressure, SAH = Subarachnoid haemorrhage.

S4: Table of all included articles

Title	Author and year	DOI	Mortality	SAH grade	Vasospasm
A segmentation-based volumetric approach to localize and quantify cerebral vasospasm based on tomographic imaging data	Neulen (2017)	10.1371/journal.pone.0172010	30.8 %	NA	NA
An apoE-derived mimic peptide, COG1410, alleviates early brain injury via reducing apoptosis and neuroinflammation in a mouse model of subarachnoid hemorrhage	Wu (2016)	10.1016/j.neulet.2016.05.058	28.6 %	13.3 (SD =2.8)	NA
Analgesic treatment limits surrogate parameters for early stress and pain response after experimental subarachnoid hemorrhage	Staib-Lasarzik (2019)	10.1186/s12868-019-0531-7	37.5 %	NA	NA
Anesthetic and subanesthetic doses of isoflurane conditioning provides strong protection against delayed cerebral ischemia in a mouse model of subarachnoid hemorrhage	Athiraman (2021)	10.1016/j.brainres.2020.147169	10.5 %	NA	69.6 % (SD = 22.0 %)
Anti-Vascular Endothelial Growth Factor Treatment Suppresses Early Brain Injury After Subarachnoid Hemorrhage in Mice	Liu (2016)	10.1007/s12035-015-9386-9	23.1 %	11.0 (1.3)	NA
Apolipoprotein E Deficiency Aggravates Neuronal Injury by Enhancing Neuroinflammation via the JNK/c-Jun Pathway in the Early Phase of Experimental Subarachnoid Hemorrhage in Mice	Wu (2019)	10.1155/2019/3832648	29.4 %	11.5 (2.5)	NA
Biglycan regulates neuroinflammation by promoting M1 microglial activation in early brain injury after experimental subarachnoid hemorrhage	Xie (2020)	10.1111/jnc.14926	21.4 %	12.9 (1.5)	NA
Calcium sensing receptor contribute to early brain injury through the CaMKII/NLRP3 pathway after subarachnoid hemorrhage in mice	Wang (2020)	10.1016/j.bbrc.2020.07.081	17.2 %	10.3 (2.2)	NA
Capillary flow disturbances after experimental subarachnoid hemorrhage: A contributor to delayed cerebral ischemia?	Anzabi (2019)	10.1111/micc.12516	61.0 %	NA	NA
Deficiency of Tenascin-C Alleviates Neuronal Apoptosis and Neuroinflammation After Experimental Subarachnoid Hemorrhage in Mice	Liu (2018)	10.1007/s12035-018-1006-z	26.7 %	10.0 (4.7)	NA

Title	Author and year	DOI	Mortality	SAH grade	Vasospasm
DHEA Attenuates Microglial Activation via Induction of JMJD3 in Experimental Subarachnoid Haemorrhage	Tao (2019)	10.1186/s12974-019-1641-y	18.4 %	NA	NA
Effect of ADAMTS-13 on cerebrovascular microthrombosis and neuronal injury after experimental subarachnoid hemorrhage	Muroi (2014)	10.1111/jth.12511	22.4 %	NA	NA
Effect of decompressive craniectomy on outcome following subarachnoid hemorrhage in mice	Buehler (2015)	10.1161/STROKEAHA.114.007703	10.0 %	NA	NA
Effects of Toll-Like Receptor 4 Antagonists Against Cerebral Vasospasm After Experimental Subarachnoid Hemorrhage in Mice	Kawakita (2017)	10.1007/s12035-016-0178-7	13.3 %	8.6 (0.9)	49.9 % (13.6 %)
Endothelial nitric oxide synthase mediates endogenous protection against subarachnoid hemorrhage-induced cerebral vasospasm	Vellimana (2011)	10.1161/STR.0b013e3182074d88	5.8 %	NA	73.4 % (24.0 %)
Endovascular Perforation Murine Model of Subarachnoid Hemorrhage	Du (2016)	10.1007/978-3-319-18497-5_14	33.3 %	NA	NA
Evaluation of a filament perforation model for mouse subarachnoid hemorrhage using 7.0 Tesla MRI	Muroi (2016)	10.1016/j.jocn.2015.10.045	90.9 %	NA	NA
Filament perforation model for mouse subarachnoid hemorrhage: surgical-technical considerations	Muroi (2014)	10.3109/02688697.2014.918579	21.1 %	NA	NA
Inhibition of AMPA (alpha-Amino-3-Hydroxy-5-Methyl-4-Isoxazole Propionate) Receptor Reduces Acute Blood-Brain Barrier Disruption After Subarachnoid Hemorrhage in Mice	Kawakita (2021)	10.1007/s12975-021-00934-0	18.5 %	8.9 (2.3)	NA
Integrated analysis of gait parameters and gene expression profiles in a murine model of subarachnoid hemorrhage	Zheng (2021)	10.1111/gbb.12728	20.0 %	NA	NA
Irisin Contributes to Neuroprotection by Promoting Mitochondrial Biogenesis After Experimental Subarachnoid Hemorrhage	Tu (2021)	10.3389/fnagi.2021.640215	18.1 %	14.7 (1.7)	NA
Long-term impairment of neurovascular coupling following experimental subarachnoid hemorrhage	Balbi (2020)	10.1177/0271678X19863021	20.0 %	NA	NA

Title	Author and year	DOI	Mortality	SAH grade	Vasospasm
Melatonin Attenuates Early Brain Injury via the Melatonin Receptor/Sirt1/NF-κB Signaling Pathway Following Subarachnoid Hemorrhage in Mice	Zhao (2017)	10.1007/s12035-016-9776-7	20.0 %	12.1 (2)	NA
Melatonin Attenuates White Matter Injury via Reducing Oligodendrocyte Apoptosis After Subarachnoid Hemorrhage in Mice	Liu (2020)	10.5137/1019-5149.JTN.27986-19.3	11.1 %	10.0 (3.1)	NA
Microthrombi Correlates With Infarction and Delayed Neurological Deficits After Subarachnoid Hemorrhage in Mice	Dienel (2020)	10.1161/STROKEAHA.120.029753	13.3 %	NA	90.4 % (9.2 %)
Minimal Long-Term Neurobehavioral Impairments after Endovascular Perforation Subarachnoid Hemorrhage in Mice	Fanizzi (2017)	10.1038/s41598-017-07701-y	28.0 %	NA	NA
Morphological Characteristics of Neuronal Death After Experimental Subarachnoid Hemorrhage in Mice Using Double Immunoenzymatic Technique	Nakano (2019)	10.1369/0022155419878181	63.4 %	6.8 (2)	NA
MRI-based in vivo assessment of early cerebral infarction in a mouse filament perforation model of subarachnoid hemorrhage	Sasaki (2017)	10.1016/j.neulet.2017.05.047	4.0 %	NA	NA
New grading system based on magnetic resonance imaging in a mouse model of subarachnoid hemorrhage	Egashira (2015)	10.1161/STROKEAHA.114.007834	19.0 %	NA	NA
Oxidative stress after subarachnoid hemorrhage in gp91phox knockout mice	Liu (2007)	10.1017/s031716710000682x	10.9 %	NA	NA
Role of the endothelium NO-Synthase in early brain injury after experimental subarachnoid hemorrhage	Lenz (2017)	10.1177/0271678X17695982	0.0 %	NA	NA
Single clip: An improvement of the filament-perforation mouse subarachnoid haemorrhage model	Peng (2019)	10.1080/02699052.2018.1531310	25.0 %	10.2 (5.5)	NA
Standardized induction of subarachnoid hemorrhage in mice by intracranial pressure monitoring	Feiler (2010)	10.1016/j.jneumeth.2010.05.005	30.0 %	NA	NA

Title	Author and year	DOI	Mortality	SAH grade	Vasospasm
Stimulator of IFN genes mediates neuroinflammatory injury by suppressing AMPK signal in experimental subarachnoid hemorrhage	Peng (2020)	10.1186/s12974-020-01830-4	17.9 %	11.0 (1.5)	NA
Subarachnoid hemorrhage in C57BL/6J mice increases motor stereotypies and compulsive-like behaviors	Nanegrungsunk (2021)	10.1080/01616412.2020.1841481	33.3 %	NA	NA
TSPO ligand Ro5-4864 modulates microglia/macrophages polarization after subarachnoid hemorrhage in mice	Zhou (2020)	10.1016/j.neulet.2020.134977	14.6 %	10.4 (1.5)	NA
Ultra-Early Cerebral Thrombosis Formation After Experimental Subarachnoid Hemorrhage Detected on T2* Magnetic Resonance Imaging	Wang (2021)	10.1161/STROKEAHA.120.032397	0.0 %	NA	NA
Value of Three-Dimensional Maximum Intensity Projection Display to Assist in Magnetic Resonance Imaging (MRI)-Based Grading in a Mouse Model of Subarachnoid Hemorrhage	Mutoh (2016)	10.12659/msm.896499	18.9 %	NA	NA
White Matter Injury After Subarachnoid Hemorrhage: Role of Blood-Brain Barrier Disruption and Matrix Metalloproteinase-9	Egashira (2015)	10.1161/STROKEAHA.115.010351	22.2 %	9.0 (3.3)	NA
White matter T2 hyperintensities and blood-brain barrier disruption in the hyperacute stage of subarachnoid hemorrhage in male mice: The role of lipocalin-2	Toyota (2019)	10.1111/cns.13221	0.0 %	NA	NA
Sevoflurane and Desflurane Exposures Following Aneurysmal Subarachnoid Hemorrhage Confer Multifaceted Protection against Delayed Cerebral Ischemia	Jayaraman (2021)	10.3390/biomedicines9070820	NA	NA	74.2 % (19.5 %)

Supplement 4: Table of included articles in meta-analysis. Articles had to report at least one of mortality, SAH grade and vasospasm. Mortality is presented as share of animals that died after SAH perforation induction. We list SAH grades exclusively that were in accordance with the scoring system proposed by Sugawara et al. (2008). Vasospasm is presented as the relative MCA, ACA, ICA and basilar artery diameter after SAH perforation induction in comparison to corresponding sham-operated mice' arteries diameter. Values in brackets represent standard deviations.
